# Coexistence of Takayasu Arteritis and Crohn's Disease in a Maltese Patient

**DOI:** 10.1155/2015/384257

**Published:** 2015-12-29

**Authors:** John Paul Caruana Galizia, Paul John Cassar

**Affiliations:** Department of Rheumatology, Mater Dei Hospital, Msida MSD 2090, Malta

## Abstract

Takayasu arteritis (TA) and Crohn's disease (CD) are uncommon chronic granulomatous disorders affecting the large arteries and the gastrointestinal tract, respectively. At least 40 occurrences of these two conditions in the same patient have been reported in the literature, raising the possibility of an association between them. We report the coexistence of TA and CD in a young Maltese patient and review the literature to discuss possible aetiological mechanisms that might explain this association.

## 1. Introduction

Takayasu arteritis is a systemic vasculitis of uncertain aetiology affecting large arteries, predominantly the aorta and its main branches. It is characterized by panarteritic granulomatous inflammation, leading to wall fibrosis and thickening, stenotic lesions, thrombus formation, and occasionally aneurismal dilation [1]. Clinical features range from asymptomatic disease discovered as a result of clinical detection of absent limb pulses to nonspecific features of fever, malaise, weight loss, arthralgia, myalgia, and anaemia to organ-specific ischaemic phenomena.

Crohn's disease is a systemic relapsing inflammatory disorder affecting predominantly the gastrointestinal tract with extraintestinal manifestations and associated immune disorders [2]. It may involve any part of the gastrointestinal tract from the mouth to the anus and is characterized pathologically by skip lesions. Clinical features include abdominal pain and diarrhoea, which may be bloody, fever, and weight loss.

Here, we report a case of coexistence of Takayasu arteritis and Crohn's disease in a teenage girl.

## 2. Case Presentation

A 17-year-old Maltese girl presented in April 2013 with a 1-week history of fever up to 100°F, night sweats, anorexia, weight loss, and abdominal pain as well as a 1-year history of loose motions with mucus but no rectal bleeding. Physical examination revealed only sparse small mouth ulcers. Investigation revealed raised inflammatory markers and folate deficiency. Stool microscopy and cultures were negative.

A colonoscopy showed patchy erythema with aphthous ulcers and skip lesions suggestive of Crohn's disease. Colonic biopsies showed oedematous colonic mucosa, a focal increased cellularity of the lamina propria with the presence of neutrophils, focal cryptitis, and occasional crypt abscesses as well as isolated superficial ulcers and ill-defined granulomas. Terminal ileal biopsies showed mucosal oedema and focal presence of neutrophils in the lamina propria but no granulomas. These biopsies were consistent with a diagnosis of Crohn's disease. She was started on prednisolone 40 mg daily, omeprazole 20 mg daily, mesalazine 1 gram tds, and calcium and vitamin D supplements.

Initially, she responded well to the treatment and prednisolone was tailed down and stopped. She maintained an ESR of 26 and a CRP of <6 and remained asymptomatic for five months. However, in January 2014, she developed abdominal pain, nausea, bloating, and anorexia and a CT enterogram was booked.

This revealed a normal terminal and small bowel with no mural thickening or hyperenhancement and no strictures and multiple enlarged mesenteric lymph nodes measuring up to 9 mm. There was circumferential mural thickening of the abdominal aorta extending over a distance of 6 cm from below the origin of the renal arteries to below the origin of the inferior mesenteric artery causing mild luminal narrowing. The celiac trunk, superior mesenteric, inferior mesenteric, and renal arteries were patent. The findings were in keeping with quiescent inflammatory bowel disease together with a newly diagnosed underlying aortitis.

At this stage, the patient complained of low back pain radiating down the left thigh as well as occasional chest pains, but there was no history of limb claudication or colour changes. She had intermittent fever and night sweats as well as episodic headaches.

**Figure 1:**
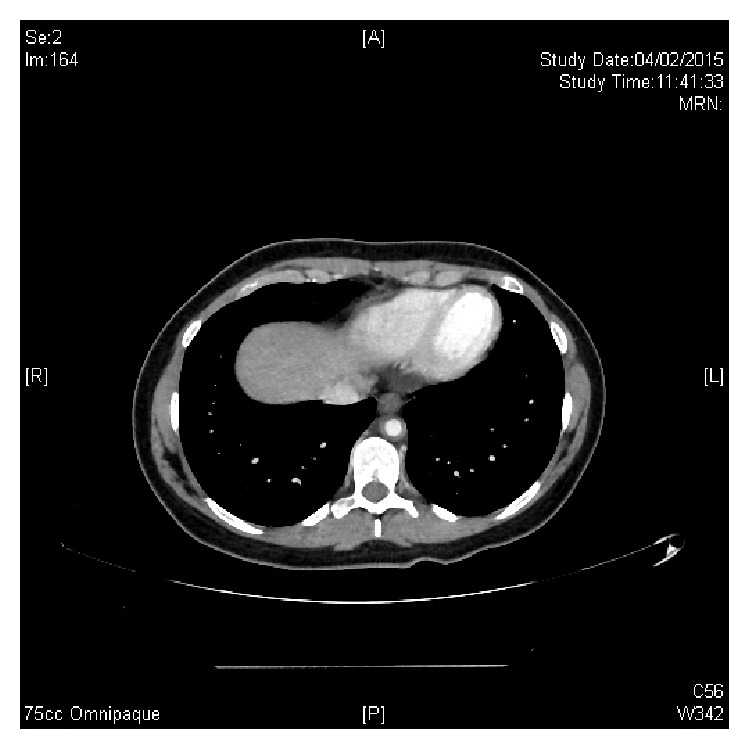
CT angiography revealing circumferential mural thickening of the lower part of the thoracic aorta.

On examination, she was afebrile and was found to have absent left radial, ulnar, and brachial pulses as well as bilateral carotid bruits more prominent on the left side. Her blood pressure was 132/78 mmHg in the right arm, but no blood pressure could be recorded on the left side. Lower limb pulses were equal bilaterally. Blood investigations were normal except for mild anaemia (Hb 10.7) and an ESR of 29. ANA, ENA, and ANCA tests were negative. A PET/CT scan confirmed the circumferential mural thickening (5 mm) starting at the origin of the left subclavian artery extending distally for approximately 3.5 cm with significant stenosis. Similar circumferential mural thickening was also present in the lower segment of thoracic aorta and in the left axillary artery and proximal part of the brachial arteries (Figure 1). The aortic arch and the rest of the main branches were normal. There was no evidence of FDG uptake along the large vessels. An MRA of the thoracic aorta was suggestive of a low-grade multifocal segmental inflammatory picture.

The patient thus met the 2006 EULAR/PRES consensus criteria [3] for diagnosis of Takayasu arteritis (see the following list) meeting the mandatory criterion as well as three of the other four criteria.

*Classification Criteria for Takayasu Arteritis*. Angiographic abnormalities (conventional CT or MR) of the aorta or major branches (mandatory criterion) plus at least 1 of the following 4 features are shown:

1. decreased peripheral artery pulse(s) and/or claudication of the extremities;
2. blood pressure difference >10 mmHg;
3. bruits over the aorta and/or major branches;
4. hypertension (related to childhood normative data).

## 3. Discussion

Both Takayasu arteritis and Crohn's disease are rare diseases. The incidence of Takayasu arteritis in adults has been reported to be 1/1,000,000/year in Europe with a female: male ratio of 1.2 : 1 [4]. No statistics relating to disease prevalence are available. The incidence of Crohn's disease is approximately 2/100,000/year and the prevalence of Crohn's disease in Europe has been reported to vary between 10 and 150 per 100,000 inhabitants [5]. The likelihood of both diseases coexisting in the same patient by chance alone has been estimated to be 1 in 10 billion individuals [6]. However, prior to this case, another 40 cases of coexistence of these two conditions have been reported in the literature since 1976 [6–9], suggesting that there may be an association between these two conditions. The mechanism of this possible association has not been yet elucidated. Common genetic susceptibilities may play a role. In this context, the MHC genes have been most extensively investigated. Takayasu arteritis has been most strongly associated with HLA class I alleles such as HLA-B52, HLA-B39, HLA-A31, and HLA-B5, whereas Crohn's disease has been more frequently associated with HLA class II alleles, though the strength of this association appears to be modest [10]. Interestingly, a recent study suggested a role for the IL-12B gene in Takayasu disease onset and progression [11]. The product of this gene (p40) is a subunit of IL-23, a cytokine that has been associated with Crohn's disease as well as psoriasis and spondyloarthropathies. The same study also suggested a link between Takayasu arteritis and the FCGR2A/FCGR3A locus [11]. This locus has been associated with other inflammatory conditions such as giant cell arteritis [12] and also, interestingly, ulcerative colitis [13, 14]. Investigation of further HLA and non-HLA gene associations in the future may yield more clues about the possible genetic link between these two diseases.
